# A molasses habitat-derived fungus *Aspergillus tubingensis* XG21 with high β-fructofuranosidase activity and its potential use for fructooligosaccharides production

**DOI:** 10.1186/s13568-017-0428-8

**Published:** 2017-06-21

**Authors:** Yijia Xie, Huanxia Zhou, Caixia Liu, Jing Zhang, Ning Li, Zhanli Zhao, Guoyong Sun, Yaohua Zhong

**Affiliations:** 1 State Key Laboratory of Microbial Technology, School of Life Sciences, Shandong University, Jinan, 250100 People’s Republic of China; 2 Shandong Xingguang Sugar Group Co. Ltd., Laoling, Dezhou, 253600 People’s Republic of China; 3 Shandong Academy of Pharmaceutical Sciences, Jinan, 250101 People’s Republic of China; 4 Anaesthesiology Department of the Second Hospital of Shandong University, Jinan, 250100 People’s Republic of China

**Keywords:** Fructooligosaccharides production, β-fructofuranosidase, *Aspergillus tubingensis*, Sugarcane molasses

## Abstract

The industrial microorganisms used for fructooligosaccharides (FOS) synthesis are generally fermented with sucrose as carbon source to induce the production of β-fructofuranosidase (FFase) having transfructosylation activity. Consequently, isolation of novel FFase producers from a sucrose-enriched biotope would help improve FOS productivity and reduce the process cost. Here, three fungi isolated from a unique sugarcane molasses habitat were found to possess FFase activity and one of them, XG21, exhibited a high capacity to synthesize FOS. Analysis of its morphological properties and ribosomal internal transcribed spacer (ITS) sequence allowed the taxonomic position to be assigned and it was thus identified as *Aspergillus tubingensis* XG21. It could utilize various potential carbon sources for vigorous growth, but only produced high-level FFase activity on sucrose. Furthermore, the transfructosylation ability and FOS synthesis were analyzed by TLC and HPLC. During the transfructosylation reaction, an increase in sucrose concentration led to the remarkable enhancement in FOS formation with the maximum content of up to 56.9% within 8 h. Finally, the sugarcane molasses was used to cultivate *A. tubingensis* XG21 and the optimal FFase activity reached up to 558.3 U/g, which was 88.9% higher than that with sucrose as carbon source. These results indicate that *A. tubingensis* XG21 can be considered as a new genetic resource adapted to cheaply available carbon sources for FOS production.

## Introduction

Fructooligosaccharides (FOS) represent a major class of prebiotics and have been classified as important functional food ingredients due to their well-recognized nutritional and health benefits (Vancova et al. 2008; Wang 2015). Chemically, FOS are short-chain fructosyl units (F) connected mainly to a terminal sucrose molecule (GF) at the reducing end. The commercial FOS preparations belong exclusively to the inulin-type and exhibit low degrees of polymerization, mainly consisting of kestose (GF2), nystose (GF3) and fructofuranosylnystose (GF4) (Dominguez et al. 2014). They have recently attracted increasing attention in the food and feed industries because of their dominant role in selectively stimulating beneficial intestinal bacteria, such as bifidobacteria (Sangeetha et al. 2005; Ishwarya and Prabhasankar 2013). At present, the major procedure for industrial FOS production is the enzymatic synthesis from sucrose by fungal β-fructofuranosidases (FFase) with high transfructosylation activity (Álvaro-Benito et al. 2007; Wang 2015). FFases are inducible enzymes and sucrose is the most well-known substrate that could induce it. However, costly media components with pure sucrose would likely render the produced enzymes too expensive for FOS synthesis. Being rich in sucrose, sugarcane molasses represents a low-cost alternative carbon source suitable for medium preparation (Miljković et al. 2016). Thus, isolation of novel FFase-producing fungi from a unique molasses habitat would contribute to develop a cost-efficient FOS production process.

Efforts on the isolation and screening of microorganisms for production of FOS-synthesizing enzymes are being made. Several fungal strains, such as *Aspergillus* sp., *Aureobasidium* sp., and *Penicillium* sp., have been reported to produce extracellular and/or intracellular enzymes with transfructosylation activity (Dominguez et al. 2014). High-activity enzymes, which have been virtually developed for industrial production of FOS, are mostly characterized from *Aspergillus* sp., especially the members belonging to the black aspergilli (*Aspergillus* section *Nigri*) (Sangeetha et al. 2005). The black aspergilli are an important group of species in food mycology and also used in the fermentation industry to produce hydrolytic enzymes, since *A. niger* used under certain industrial conditions has been generally recognized as safe (GRAS) status by the US Food and Drug Administration (FDA) (Varga et al. 2011). It has been known that *A. niger* and *A. tubingensis* are the dominant (Mirhendi et al. 2016). Although the ability of *A. niger* to produce high-level FFases with transfructosylation activity has been described (Hidaka et al. 1988; Wang and Zhou 2006; Yanai et al. 2001), the species *A. tubingensis* has not been reported to be a FFase producer. Various reports evidenced that members of the *A. tubingensis* species are among the commonest fungi isolated from diverse habitats including some important agricultural products and are frequently responsible for post-harvest decay of fruits and some vegetables (Perrone et al. 2007). In particular, a novel polymeric material (specifically, polyester polyurethane, PU) degrading fungus was recently isolated from the soil of a city waste disposal site and identified as *A. tubingensis* (Khan et al. 2017). These findings highlight the ability of the *A. tubingensis* species to adapt to environmental conditions.

In this study, a FOS-synthesizing fungal strain *A. tubingensis* XG21 was isolated from a unique molasses habitat. The transfructosylation reaction using the whole cells of this strain under different concentrations of sucrose was determined. This is the first report of the characterization of a *A. tubingensis* strain for FOS production. Furthermore, the high FFase activity was achieved using the sugarcane molasses as the carbon source for fermentation of *A. tubingensis* XG21.

## Materials and methods

### Culture media

The Czapek Dox (CD) medium consisted of sucrose 30 g/L, NaNO_3_ 3 g/L, K_2_HPO_4_ 1 g/L, MgSO_4_·7H_2_O 0.5 g/L, KCl 0.5 g/L, FeSO_4_ 0.01 g/L and CuSO_4_ 0.01 g/L. The potato dextrose agar (PDA) medium was composed of peeled potato extract 200 g/L, glucose 20 g/L and agar 20 g/L. The fungal minimal medium (MM) was prepared as described by Penttilä et al. (1987). The fermentation medium (FM) contained sucrose 80 g/L, yeast extract 15 g/L, KCl 0.5 g/L, MgSO_4_·7H_2_O 0.5 g g/L, K_2_HPO_4_ 5 g/L, NaNO_3_ 2 g/L.

### Isolation and preliminary screening of β-fructofuranosidase producing fungi

Different soil samples were collected from Shandong Xingguang Sugar Group Co. Ltd., P. R. China, a private enterprise engaging in extracting sucrose from sugar-cane with a history of nearly 20 years. To isolate fungal strains, the soil samples were serially diluted and spread on CD agar plates mentioned above and incubated at 30 °C for 3 days. The cultures were purified by repeated transfer to new fungal MM agar plates. Then the single colonies were grown in PDA plates at 30 °C for 6 days. The spores were washed with sterilized physiological sodium chloride solution. To rapidly determine the ability of these fungi to produce FFase, an indirect colorimetric plate assay for evaluation of FFase was used, which involved in the detection of glucose released from sucrose. This method entailed a glucose oxidase–peroxidase (Dingguo Corp., Beijing, China) coupled reaction using phenol and 4-aminoantipyrine for determination of glucose with the formation of pink halos around the fungal colony (Dominguez et al. 2006). In detail, 1 μL of spores (10^8^/mL) was spotted on the CD agar plates and incubated at 30 °C for 8 h. Then 10 mL of the biochemical reaction system, composed of glucose oxidase (GOD, 10 U/mL), horseradish peroxidase (POD, 1 U/mL), 4-aminoantipyrine (0.16 mg/mL) and phenol (1 mg/mL) in the citric phosphate buffer (pH 5), was placed on the plates. After 10 min, the formation of pink halos around the colonies indicated the ability of the fungi to produce FFase.

### Secondary screening of the FFase producers with transfructosylation activity

The spore suspensions (10^8^ spores) of the candidate fungi were inoculated into 30 mL of FM media. After incubation at 30 °C for 48 h, the cultures were centrifuged and the cells were collected for rapid FOS synthesis reaction. The reaction mixture (5 mL) consisted of 25% (W/V) sucrose (2 mL) as the substrate, 0.1 M citrate buffer (pH 5.0, 3 mL) and adequate amount of the collected cells. The enzymatic reaction was carried out at 50 °C for 2 h with moderate shaking and terminated by heating the mixture in boiling water for 10 min. Then qualitative identification of FOS production and composition was conducted using the thin layer chromatography (TLC) according to the method described by Zhang et al. (2017).

### Morphological classification and molecular identification

The taxonomic assignment of the target fungus was followed for observation and identification. Macroscopical characters were assessed at 6 days on PDA plates. To determine the microscopic characteristics of the fungus, 1 μL of spores (10^8^/mL) were inoculated on CD plates and the glass cover slips were inserted 2 cm far from the spores. After incubation at 30 °C for 3 days, the slips were used for observation of the conidial heads, conidiophores and conidia shapes under a microscope. Each strain was identified in genus level according the standard methods provided by Pitt and Hocking (2009). Experiments were conducted thrice with 3 replicate plates.

The fungal mycelia were harvested from MM medium after cultivation of 3 days at 30 °C. Then the total genomic DNA was extracted using the DNeasy Plant Mini-Kit (Qiagen, Valencia, CA, USA). The internal transcribed spacer (ITS) sequence of fungal nuclear ribosomal DNA was amplified by PCR using ITS-F (5′-TCCGTAGGTGAACCTGCGG-3′) and ITS-R (5′-TCCTCCGCTTATTGATATGC-3′) as primer pair. The PCR product was separated by electrophoresis and then isolated and sequenced. The fungal ITS sequence was deposited into GeneBank Data Library with the accession number KY705016. A homology search was performed with GenBank database and the homologous sequences were selected for phylogenetic analysis by using the neighbor-joining (NJ) method. Then an NJ tree was constructed using MEGA version 5.0. A bootstrap analysis was performed with 1000 replications as confirmation of each clade.

### Fungal growth and FFase production on different carbon sources

10^8^ spores were inoculated in 100 mL of CD liquid media with 3% sucrose replaced by 1% sucrose, maltose, glucose, fructose, xylose or glycerol as carbon sources. The cultures were taken out every 12 h to measure dry weight of mycelia and FFase activity. The FFase activity was determined according to the method of Hidaka et al. (1988). One unit of activity (U) was defined as the amount of enzyme required to produce 1 μmol of glucose per min. To further investigate the ability of the fungus to produce FFase on sucrose, 10^9^ spores were inoculated in 300 mL of FM containing 8% sucrose as carbon source. The cultures were taken out every 12 h to measure dry weight of mycelia, supernatant pH and FFase activity.

### Transfructosylation reaction and chromatography analysis

For FOS synthesis, a reaction mixture consisting of 20% sucrose (w/v) and fungal cells (6 units of enzyme per g sucrose) in 50 mM citrate phosphate buffer (pH 5.5) was stirred at 50 °C. The mixture was taken at appropriate times and treated in boiling water for 5 min to terminate the reaction. Then the sample was centrifuged at 1, 600 g for 10 min. The supernatant was collected and subjected to TLC to examine the homogeneity of the resulting products. To further analyze the effects of substrate concentration on the transfructosylation activity, different concentrations of sucrose (w/v) (0.2, 2, 5, 20 and 50%) were used for FOS synthesis. Quantitative analysis of the reaction products was carried out using high-performance liquid chromatography (HPLC, LC-6A, Shimadzu, Japan) with a Agilent Zorbax NH_2_ column (5 μm, 4.6 mm × 250 mm) (Agilent Technologies, Santa Clara, CA) coupled with a refractive index detector (2414, Waters, USA). The mobile phase was acetonitrile: water (70:30, v/v) at a flow rate of 1.0 mL/min. The identification and quantification of each FOS was carried out by a calibration curve built with authentic standards of kestose, nystose and fructosyl nystose (Sigma Corp., St Louis, MO).

### Production of FFase using sugarcane molasses as carbon source

For inoculation, 10^9^ spores were added into 300 mL of the culture medium and cultivated at 30 °C and 200 rpm. The culture medium consisted of the basal medium and various amounts of sugarcane molasses [2, 5,10, 15 and 20% (w/v)]. The basal medium contained yeast extract 15 g/L, KCl 0.5 g/L, MgSO_4_·7H_2_O 0.5 g/L, K_2_HPO_4_ 5 g/L, NaNO_3_ 2 g/L. After incubation for 48 h, the cells were collected and examined for the FFase activity. All experiments were conducted in triplicate.

## Results

### Screening for fungal strains with FFase activity

**Fig. 1:**
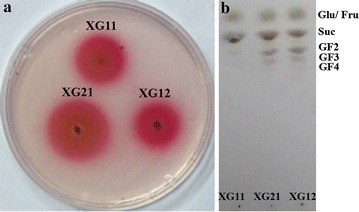
Screening of fungal strains based on their ability to produce FFase and synthesize FOS. **a** Detection of glucose released from sucrose around the fungal colonies grown in the CD plate by a glucose oxidase–peroxidase coupled reaction. XG11, XG21 and XG12 are the fungal strains obtained from the sugarcane molasses habitat. **b** TLC analysis of the resulting saccharides from the transfructosylation reaction by the fungal strains XG11, XG21 and XG12. *Glu* glucose; *Fru* fructose; *Suc* sucose; *GF*_*2*_ kestose; *GF*_*3*_ nystose; *GF4* fructofuranosyl nystose

To obtain the FFase-producing fungi from a unique molasses habitat, the Czapek Dox agar plates (CD) was firstly applied for cultivation of various samples taken from a professional sugar manufacturing company with a lengthy history in Shandong province, China. It is known that glucose and fructose are the reaction products accompanying FOS synthesis and the excess of glucose over fructose is characteristic of the transfructosylation activity of FFase (Chen and Liu 1996). Thus, the detection of glucose by using the glucose oxidase (GOD)-peroxidase (POD) system could provide a rapid and indirect assay for screening for potential candidate FOS-synthesizing fungi (Dominguez et al. 2006). Here, the isolated fungal strains were tested with the biochemical GOD-POD reaction system. Three strains with the large pink halos around their colonies, namely XG11, XG12 and XG21, were selected (Fig. 1a). To further confirm the transfructosylation activity of these fungi, thin-layer chromatography (TLC) was use to analyze the reaction products after 3 h incubation in 25% sucrose with the fungal cells (Fig. 1b). The results showed that XG12 and XG21 could produce clear bands of fructooligosaccharids (GF2, GF3 and GF4) while XG11 didn’t exhibit the corresponding bands on the chromatography plate, indicating the various transfructosylation activities among the selected fungal strains. Since XG21 showed the largest pink halos around the colony and the relatively higher amount of fructooligosaccharids after transfructosylation reaction, it was selected as the target strain for further study.

### Identification of XG21 by morphological classification and phylogenetic analysis

**Fig. 2:**
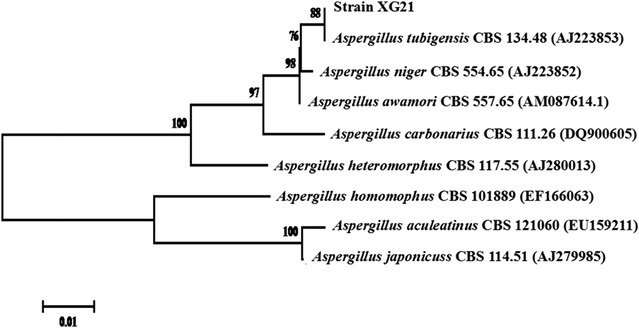
Phylogenetic tree showing the position of the strain XG21 based on the internal transcribed spacer (ITS) sequence of nuclear ribosomal DNA. The number at the branch nodes indicates bootstrap values (%) based on 1000 replications. The *scale bar* denotes 0.01 changes per nucleotide position

The cultural and microscopic characteristics of the isolate XG21 were analyzed to classify the fungal genus with the key evidence-based evaluation (Jung et al. 2012; Pitt and Hocking 2009). The colonies and microscopic morphological characters were shown in Fig. 2. XG21 exhibited radial mycelial growth, typical black sporulation layer and white mycelia at the edge of each colony on PDA plate. Microscopic examination showed the presence of upright conidiophores terminating in a swollen vesicle, with many conidiospores on it, which is typical for *Aspergillus* sp. (Trivedi et al. 2012). The results suggested that the isolate XG21 probably belongs to the genus *Aspergillus*.

**Fig. 3:**
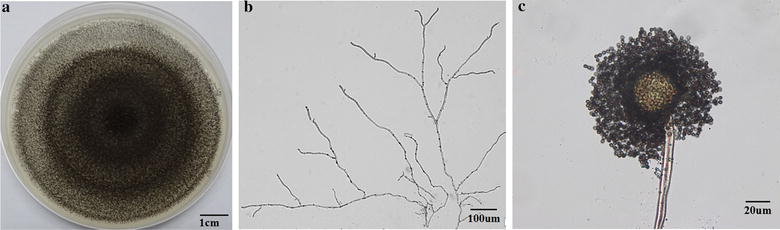
Morphological characteristics of *A. tubingensis* XG21. **a** Growth on PDA medium at 30 °C for 6 days. *Scale bar* 1 cm. **b** Microscopic observation of the XG 21 mycelium grown on CD plate. *Scale bar* 100 μm. **c** Conidiophore and conidia. *Scale bar* 20 μm

The amplified nuclear ribosomal internal transcribed spacer (ITS) region of the strain XG21 was sequenced and compared with the ITS sequences of micro-organisms represented in the NCBI database gene bank using BLAST. The BLASTn result for its ITS sequence showed 100.0% sequence identity to those of the *A. tubingensis* strains. A neighbor-joining phylogenetic tree was constructed by using the ITS sequences of XG21 and other closely related fungal strains according to the GenBank database, which are the currently accepted species of *Aspergillus* section *Nigri* (Varga et al. 2011). From the ITS phylogenetic analysis, XG21 shared the same latest common origin and evolutionary lineage with *A. tubingensis* and followed by *A. niger* (Fig. 3). On the basis of the ITS sequence analysis, together with its morphological characteristics, the strain XG21 was identified as *A. tubingensis* and hence named *A. tubingensis* XG21. To date, there is no report on the ability of synthesizing FOS by *A. tubingensis* and so *A. tubingensis* XG21 was used for analysis of carbon source-dependent growth and FFase production.

### Effects of various carbon sources on cell growth and enzyme production

**Fig. 4:**
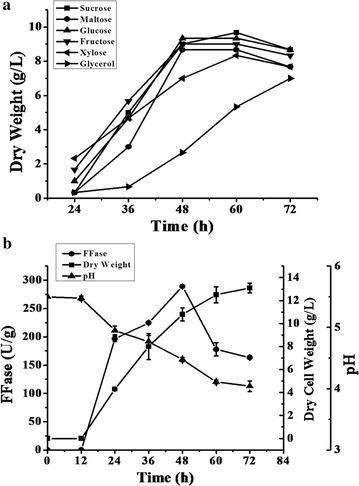
Growth and FFase production of *A. tubingensis* XG21 on different carbon sources. **a** The fungal growth *curves* by using 1.0% sucrose, maltose, glucose, fructose, xylose or glycerol as carbon sources. **b** Kinetics of FFase production by *A. tubingensis* XG21 grown in 8.0% sucrose as carbon source

The growth pattern of *A. tubingensis* XG21 was observed for three days with different carbon sources (Fig. 4a). When sucrose, maltose, glucose or fructose was used, excellent growth was achieved, reaching a maximum of approx. 10 g/L. Growth on xylose or glycerol was relatively poor, especially the latter exhibiting the lowest growth of about 6 g/L. When the fungal cultures from different carbon sources were taken for the assay of FFase activity, only the sucrose-grown culture possessed high enzymatic activity (data not shown). Therefore, the kinetics of the enzyme production of *A. tubingensis* XG21 by using 8% sucrose as the sole carbon source was investigated (Fig. 4b). Before reaching a maximum, the enzyme activity increased at a rate proportional to the increase in cell growth. By contrast, a sharp decrease in pH, from 5.5 to 4.1, is observed from 12 to 60 h and then keep more or less constant toward the end of cultivation (Fig. 4b). The activity of FFase was the greatest at 48 h during the exponential growth stage and reached 295.6 U/g (Fig. 4b). These results indicated that *A. tubingensis* XG21 is a potential strain for FOS synthesis due to its favorable FFase activity.

### Determination of the behavior of FOS formation

**Fig. 5:**
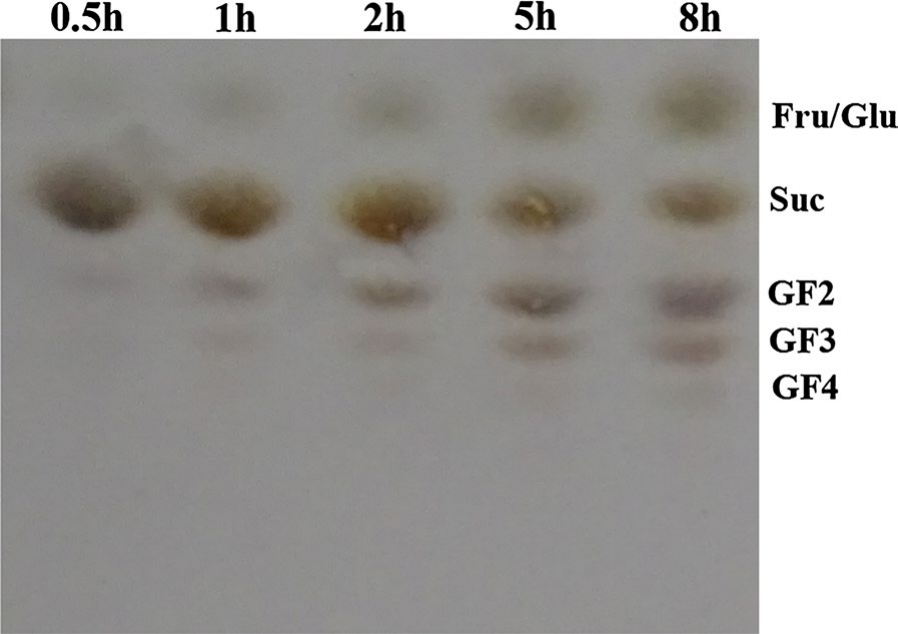
TLC analysis of the FOS composition formed during the transfructosylation reaction using 20% sucrose as substrate. *Glu* glucose; *Suc* sucose; *Fru* fructose; *GF*_*2*_ kestose; *GF*_*3*_ nystose; *GF4* fructofuranosyl nystose

Evaluation of FOS formation with 20% (w/v) sucrose as substrate was firstly studied at different time intervals (Fig. 5). It was found that kestose (GF2) appeared at 0.5 h while nystose (GF3) and fructofuranosyl nystose (GF4) began to form at 1 and 5 h, respectively. Notably, the highest kestose formation was observed at 5 h of reaction and then decreased gradually as the reaction progressed. Furthermore, the effects of sucrose concentrations on the reaction products were investigated by detecting the changes of the carbohydrate composition in different sucrose concentrations ranging from 0.2 to 50% (Table 1). It was discovered that the increase in sucrose concentration led to the remarkable enhancement in FOS formation from 1.0 to 56.9% of the carbohydrate composition, while fructose decreased sharply from 44.5 to 1.7%. More specifically, the reaction with the lowest substrate concentration (0.2%) monitored in the study afforded a mixture of almost completely hydrolyzed products, glucose (49.2%) and fructose (44.5%).

**Table 1:** HPLC analysis of FOS yield and composition produced by the FFase of *A. tubingensis* XG21 at different substrate concentrations

Substrate (w/v) (%)	Sucrose (%)	Glucose (%)	Fructose (%)	GF2 (%)^b^	GF3 (%)^b^	GF4 (%)^b^	GFn (%)
0.2	5.4 (±0.2)^a^	49.2 (±2.3)	44.5 (±3.1)	1.0 (±0.2)	0.0 (±0.0)	0.0 (±0.0)	1.0
2.0	16.5 (±2.6)	44.9 (±1.2)	24.9 (±4.2)	8.3 (±0.3)	4.9 (±0.3)	0.5 (±0.1)	13.7
5.0	21.0 (±1.7)	40.8 (±2.1)	17.6 (±1.3)	10.2 (±1.2)	8.6 (±0.8)	2.9 (±0.2)	21.7
20.0	18.5 (±3.2)	37.9 (±1.9)	5.3 (±0.6)	13.9 (±0.9)	19.1 (±2.2)	5.4 (±1.2)	38.4
50.0	8.2 (±1.2)	31.3 (±3.2)	1.7 (±0.2)	21.8 (±2.2)	30.5 (±2.3)	6.6 (±1.7)	56.9

The enzyme dosage was 6 U/g sucrose (substrate) and the reaction was conducted at 50 °C for 8 h

^a^ Data are the mean of three independent experiments; values in parentheses show standard deviations

^b^ *GF2* kestose; *GF3* nystose; *GF4* fructofuranosyl nystose

### Improvement of FFase production using sugarcane molasses as carbon source

**Fig. 6:**
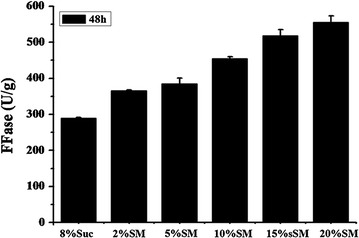
Production of FFase by *A. tubingensis* XG21 using sugarcane molasses (SM) as carbon source. Different amounts of sugarcane molasses (2, 5, 10, 15 and 20%) were used to cultivate *A. tubingensis* XG21 and the fungal cells were collected after 48 h to examine the FFase activity. 8% sucrose (Suc) was used as the control carbon source for fungal growth. All experiments were conducted in triplicate

Considering that *A. tubingensis* XG21 was isolated from a unique sugar-manufacturing habit, sugarcane molasses could be used as a promising alternative nutritional source for the fungal growth with the cost reduction and the enzyme production improvement. Thus, the cheaply available sugarcane molasses was used to cultivate this fungus for FFase production (Fig. 6). It was found that the lowest concentration (2%) of sugarcane molasses used in this study could provided higher FFase production (365.8 U/g) than 8% sucrose (295.6 U/g). Furthermore, the increase of sugarcane molasses concentration resulted in a linear increase in enzymatic activity and the optimal enzyme production reached up to 558.3 U/g (calculated as 62.3 U/mL) at 20% sugarcane molasses concentration (that is, corresponding to 8% sucrose), which was 88.9% higher than the enzyme production with 8% sucrose as carbon source.

## Discussion

Commercially, FOS can be produced from sucrose using microbial FFases with high transfructosylation activity. Fungal strains, especially those from the *Aspergillus* genus, are good FOS producers (Dominguez et al. 2014; Hirayama et al. 1989). However, these industrial strains are generally accustomed to sucrose as the carbon source for FFase production, which is uneconomical for large-scale fungal fermentation processes. Here, a novel fungus, *A. tubingensis* XG21, was isolated from a sugar-manufacturing habitat and characterized with the remarkable ability to synthesize FOS. Transfructosylation reaction showed that the FFase produced by this strain had high transfructosylation activity. Especially, *A. tubingensis* XG21 exhibited superior adaptability to the cultivation condition with sugarcane molasses as carbon source and could produce high-level FFase.

The carbon source of the culture medium is one of the most important factors influencing cell growth and physiology, and hence bioproduct formation. Vainstein and Peberdy (1991) reported that the growth of another FFase-producing fungus *A.nidulans* was strongly influenced by the carbon sources, among which sucrose and glucose supported the maximum fungal growth. Similarly, sucrose, maltose, glucose and fructose were found to be the preferential carbon sources for growth of *A. japonicus* MU-2, while glycerol showed the slowest growth (Hayashi et al. 1992). The similar phenomenon was also found in *A. japonicus* TIT-90076, a famous FOS synthesizing fungus with high transfructosylation activity (Chen and Liu 1996). In this study, the difference in fungal growth observed Fig. 4a could be attributed to the fact that glucose and fructose, other than xylose and glycerol, are the easily metabolisable carbon sources as suggested by the previous studies (Narasimha et al. 2006). Nevertheless, the fast fungal growth of *A. tubingensis* XG21 on sucrose or maltose indicated the occurrence of adequate sucrose/maltose-metabolizing enzymes in this fungus.

Furthermore, sucrose was shown to be the effective inducer for FFase production from *A. tubingensis* XG21 (Fig. 4b), which was in accordance with the previous report that the β link and the fructose located at the end of the molecule are involved in the induction mechanism (Rubio and Navarro 2006). The activity of FFase was the greatest at 48 h during the exponential growth stage and fell progressively with increasing time (Fig. 4b). These results were similar to FFase production by *A. nidulans* (Eidam) 2.1, *A. japonicus* TIT-90076 and *A. niger* GH1 (Vainstein and Peberdy 1991; Chen et al. 1996; Veana et al. 2011). In these cases, the decrease of FFase activity seemed to be related to the depletion of the carbon source for mycelium growth and the decline of pH in the culture media. The maximum FFase activity produced by *A. tubingensis* XG21 reached 295.6 U/g (Fig. 4b). Such a value compares well with other activities for the commercial FOS producers such as *A.niger* ATCC 20611 (Hidaka et al. 1988), *A. japonicus* ATCC 20236 (Mussatto et al. 2009) and *Aureobasidium pullulans* DSM2404 (Yoshikawa et al. 2006) and exceeds the the activities for some recently isolated fungi including *A. oryzae* IPT-301 (Fernandez et al. 2007), *A. niger* GH1 and *Penicillium pinophilum* EH2 (Veana et al. 2011). Therefore, *A. tubingensis* XG21 is considered to be a potential strain for FOS synthesis due to its favorable FFase activity.

It was reported that some fungi-derived FFases had the ability to catalyze fructosyl transfer to produce oligosaccharides, whose amounts could increase with increasing substrate concentration (Hirayama et al. 1989). In this study, kestose (GF2) was the first-formed product in initial reaction time (0.5 h) while the higher molecular weight of oligosaccharides, nystose (GF3) and fructofuranosyl nystose (GF4), began to form at later reaction stages. The decrease of the kestose content in late reaction period can be attributed to the fact that it serves as acceptor for further oligomerization to synthesize nystose and 1-fructofuranosyl nystose (Dominguez et al. 2014; Rubio and Navarro 2006). On the other hand, the enzyme reaction in 50% sucrose solution yielded the FOS that was almost entirely accompanied by glucose as the by-product (Table 1). These results indicated that the FFase produced by *A. tubingensis* XG21 had a strong transfructosylation activity at high substrate concentration. It is worth mentioning that the pattern of FOS formation by *A. tubingensis* XG21 was similar to several other FOS-producing fungi (Hidaka et al. 1988; Shin et al. 2004). Nevertheless, the amount of GF2 formed at the end of reaction by *A. tubingensis* XG21 was less than those with the famous industrial fungus *A. niger* ATCC 20611 and other newly isolated *Aspergillus* species (Hidaka et al. 1988; Ganaie et al. 2013), showing its high transfructosylation activity. These results indicated that this peculiar fungus isolated in this study would be valuable for industrial process due to the reduction in the time needed for FOS synthesis.

Sucrose is the most commonly used carbon source for commercial production of FFase and fungal biomass, which are further applied for FOS synthesis (Chen and Liu 1996; Dominguez et al. 2014; Hidaka et al. 1988). However, the use of purified sucrose as substrate is uneconomical for large scale cultivation of industrial fungi. The use of agro-industrial wastes or alternative carbon sources for production of FFase has been reported previously. For example, the highest level of intracellular FFase (26.58 U/mL) was obtained by growing *A. niveus* with sugarcane bagasse as carbon source, which was slightly higher than that (24.74 U/mL) with sucrose (Guimarães et al. 2009). Another fungus *A. niger* PSSF21, which was isolated from agricultural fields, was reported to produce maximal FFase at only 19.1 U/mL using sugarcane molasses as carbon source (Reddy et al. 2010). More recently, a newly fungus *A. versicolor* isolated from Atlantic Forest-Brazil could produce 48.17 U/mL FFase using apple pomace as carbon source after 8 d cultivation under the optimized condition (Dapper et al. 2016). These results showed that the low-cost carbon sources could be used for production of FFase and hence reduce the FOS production cost. In this study, *A. tubingensis* XG21 exhibited the FFase-producing capacity superior to those strains mentioned above under the cultivation conditions with cheap substrates as carbon sources. This is probably due to the remarkable adaptability of XG21 to the molasses habitat. These results demonstrated that the fungus *A. tubingensis* XG21 has the great potential for industrial FOS production, and also illustrated the feasibility of access to microbial genetic resources used for synthesizing valuable bioproducts by precisely isolating microorganisms from special habits.

## Abbreviations

FOS: fructooligosaccharides
FFase: β-fructofuranosidase
*A. tubingensis*: *Aspergillus tubingensis*
CGMCC: China General Microbiological Culture Collection Center
TLC: thin layer chromatography
HPLC: high performance liquid chromatography
GOD: glucose oxidase
POD: peroxidase
